# Can Prenatal Services Learn from Pregnancy Outcomes? A 16-Year Feasibility Study of Data Availability Across Fragmented Maternity Care

**DOI:** 10.3390/healthcare14203526

**Published:** 2026-10-20

**Authors:** Maria Cezara Mureșan, Marius Bogdan Mureșan, Dan Dumitrașcu Biriș, Zoran Laurențiu Popa

**Affiliations:** 1 Department of Obstetrics and Gynecology, Faculty of Medicine, Victor Babeș University of Medicine and Pharmacy, 300041 Timișoara, Romania; 2 Maternal-Fetal Medicine Center, 300236 Timișoara, Romania

**Keywords:** routinely collected health data, obstetric registry, data availability, outcome ascertainment, intrapartum compromise, cord blood gases, longitudinal care, feasibility

## Abstract

Background/Objectives: Prenatal services generate increasingly dense antenatal data, but their ability to evaluate what those data predict depends on outcome information arising in delivery services they may not control. Routinely collected data may fail to support a longitudinal clinical study for fundamentally different reasons: the required event may never occur, the relevant quantity may never be measured, or information generated elsewhere may never reach the record. Record linkage addresses only the last of these. In a retrospective descriptive feasibility and data-availability study, we assessed whether the information required for an intended longitudinal obstetric study could be reconstructed from routinely collected records, distinguishing these mechanisms and examining how availability differed across 16 years of index examinations. The intended biological hypothesis was not tested. Methods: We analysed routinely collected health data from the obstetric clinical database of a private maternal–foetal medicine centre in Romania, comprising 9008 administrative keys from 8090 women, with index first-trimester examinations from 11 August 2010 to 31 August 2026. Record-level linkage to the source export was verified in a 500-record sample. Delivery-data availability was assessed in records that had passed a gestational-age-derived late-gestation reference date plus a 90-day lag before the analysis cutoff, with 0- and 180-day sensitivity analyses, and it was compared descriptively across four calendar epochs. We distinguished observations not generated, not measured and not documented, and we report a sensitivity analysis restricted to one key per woman. Results: Among 8436 outcome-mature keys, availability of examinations in both later gestational windows rose from 431/1631 (26.4%) in 2010–2014 to 514/1149 (44.7%) in 2024–2026, while documentation of delivery mode fell from 932/1631 (57.1%) to 123/1149 (10.7%), in absolute as well as relative terms. The divergence persisted under both alternative maturity lags and when each woman contributed a single key (26.0% to 44.1%, and 56.3% to 9.6%). Mean arterial pressure, a measurement made within the service, rose from 0.06% to 95.2% over the same period. Within the contracting subset with a documented mode, recorded indications became more frequent (1.7% to 42.3%), so the composition of the information that returned changed as its volume fell. Cord blood gases were an investigator-confirmed structural zero within the study pathway and the analysed export contained no field for the 5 min Apgar score, so the intended acid–base-supported endpoint could not be reconstructed. All proportions describe documentation present in the analysed export at extraction, not the occurrence of clinical events. Conclusions: Evaluating prenatal prediction requires adequate ascertainment of the relevant pregnancy outcomes, and whether that condition holds is an empirical question for each service. Increasing antenatal observation did not ensure continuity of delivery information, and the composition and informational content of the outcomes that did return also changed. Documentation failure arose both from fields left empty and from fields the record did not contain. Beyond research, inadequate outcome ascertainment may limit a service’s ability to audit and evaluate its own prenatal screening performance. These data do not test the biological hypothesis; they identify requirements for a valid future test.

## 1. Introduction

A longitudinal biological question can require information that no single part of the care pathway routinely produces. In obstetrics, a first-trimester assessment, later foetal examinations, labour management and neonatal evaluation may occur in different institutions. An antenatal record can therefore be detailed while remaining insufficient to connect early observations with an interpretable delivery endpoint. The relevant question is whether the required sequence can be reconstructed without changing its clinical meaning.

The intended study originated from a proposed relational framework of placental sufficiency, in which foetal adequacy depends on the balance between changing demand and effective maternal–placental supply [1]. The framework motivated the hypothesis that reduced functional reserve could remain clinically unapparent during pregnancy and become apparent under the physiological stress of labour, including in foetuses not classified as growth-restricted. The monograph is cited solely to explain the logic and origin of this hypothesis. Its propositions are not treated as established evidence or validated by the present analysis.

Independent clinical literature supports the need to distinguish foetal oxygenation during labour from the response to antenatal conditions [2]. Contemporary intrapartum guidance interprets foetal heart-rate patterns within their clinical context and uses explicit monitoring classifications [3,4]. A retrospective test of labour tolerance would consequently require more than a delivery-mode label: actual labour exposure, interpretable evidence motivating intervention, and a neonatal endpoint with a defined relationship to the intrapartum process would be needed.

Where prenatal assessment and delivery occur in different parts of a health system, other groups have had to recover outcome information deliberately rather than expect it to arrive. The principle is long-established: an audit of prenatal serum screening and diagnostic testing across four London maternity units linked obstetric records, screening referrals and prenatal diagnosis data for 110,272 patients and concluded that continuing audit of prenatal programmes could be derived from computerised maternity datasets, provided these included foetal deaths and the relevant laboratory and ultrasound information [5]. More recently, a feasibility study of screening-biomarker evaluation obtained birth outcomes for 1681 of 1882 women (89.3%) through record linkage to routinely held birth data and concluded that linkage is an efficient route to evaluating predictive tests in pregnancy [6]. At programme scale, the evaluation of detection rates for a national foetal anomaly screening programme required active multi-source ascertainment, linkage, audit and validation across 12,694 diagnoses [7]. Such programmes illustrate what a closed information loop looks like; a service without that infrastructure occupies the other end of the same spectrum.

Record linkage, however, addresses one specific failure: information that exists somewhere and has not reached the analysed record. It cannot recover a physiological exposure that never occurred, nor a measurement that was never performed anywhere. Distinguishing these situations therefore determines which fraction of an apparent data deficit is recoverable at all, and by what means. That distinction is the contribution of the present study.

Routinely collected records are produced for care and administration rather than for a particular research protocol. RECORD extends STROBE for studies using routinely collected health data by requiring explicit reporting of database provenance, population-selection algorithms, data-cleaning procedures, and limitations arising from the use of data not originally collected for the research question [8,9]. Data-quality frameworks also distinguish completeness from conformance and plausibility, and they separate internal verification from validation against an external reference [10]. A recent overview of reviews maps the proliferation of such frameworks and the absence of consensus on the terminology, definitions and assessment methods used for data-quality dimensions [11]. These distinctions matter when an absent observation may reflect an event that did not occur, a measurement that was not undertaken, or information that did not reach the database.

The present study examines the feasibility of the intended biological investigation in one defined administrative cohort. Its objectives were to determine which required observations were available, distinguish the mechanisms limiting their availability, and compare antenatal observation with delivery-data capture across calendar epochs. A further objective was to assess whether indication documentation among captured outcomes changed in parallel with overall capture. We did not estimate associations involving first-trimester marker values or analyse foetal-weight values or trajectories.

## 2. Materials and Methods

### 2.1. Design and Setting

We conducted a retrospective descriptive feasibility study using routinely collected records from a private maternal–foetal medicine centre in Romania. The centre provides first-trimester combined screening and specialist foetal assessment; it does not provide delivery care. Births therefore occurred in other institutions, and delivery information depended on its subsequent return to the antenatal record. The dataset did not provide a complete population-based register of births or a verified history of information transfer between institutions.

Patients attending this antenatal service formed a self-selected population. The clinical investigators note that women able to obtain private foetal-medicine assessment constitute a distinct group that does not correspond to either the public or the private delivery sector: many subsequently deliver within public maternity units while differing from the general population served by those units. The database records neither socioeconomic characteristics nor the institution of delivery, so this observation is reported as investigator-supplied context and is not derived from the analysed data. It is relevant because delivery-mode composition in this cohort should not be read as describing the practice of the centre, which performs no deliveries, nor as an estimate for either national sector.

All administrative keys meeting these eligibility criteria within the analysis period were included, without additional sampling; the cohort is therefore consecutive with respect to the stated rules. The export records neither referral source nor socioeconomic characteristics, so we cannot describe whether referral patterns or patient composition changed during the period. This limitation is relevant to the interpretation of the temporal comparisons and is restated in Section 4.7.

Outcomes return to the antenatal record. Because the centre does not provide delivery care, information about the delivery could reach the antenatal record only after the pregnancy had ended elsewhere. The investigators describe the routes in use during the study period: a patient follow-up form introduced in 2011; an institutional email address through which patients could return information; manual entry of returned information into the clinical record by nursing staff; completion of an earlier pregnancy’s fields when a woman reattended for a subsequent pregnancy; reminders; and telephone contact when returned information was incomplete or internally inconsistent. Separately, selective telephone retrieval has been undertaken for defined research projects addressing particular calendar years or conditions, each under its own ethics approval; those projects began recently and remain in progress. None of these routes was automated, and none depended on transfer of records from the delivering institution. Taken together, they did not constitute uniform active ascertainment of the historical cohort.

For outcome fields, the analysed export retained neither the source of the information nor the date on which it was received or entered. Three consequences follow. First, the presence of an outcome value on an antenatal source row does not establish that the outcome was known at the time of that examination. Second, the contribution of each retrieval route cannot be quantified and reporting delay cannot be estimated. Third, because a record continues to accumulate opportunities for retrieval for as long as it remains in the database, the time available for outcome information to arrive is not equal across index years; this is addressed in Section 3.4 and Section 4.4. Historical completion of the database is distinct from the present secondary analysis: the statement that no patient was contacted applies to this analysis and does not describe how the database was completed in preceding years.

Analysis period. The analysis dataset was restricted to index first-trimester examinations performed from 11 August 2010 to 31 August 2026 inclusive, so as to remain within the data period covered by the framework ethics approval. This restriction removed 19 records with index examinations dated 1–10 September 2026; none had a second- or third-trimester examination, a recorded delivery mode, birthweight, delivery indication or pre-eclampsia entry, and the restriction therefore did not alter any reported availability cascade. The end of the approved data period is fixed by the ethics documents and not by any property of the records; the accompanying check on those 19 records was performed afterwards and is reported only to show that the restriction did not alter any reported cascade. The analysis cutoff of 31 August 2026 is the latest observation date used for the maturity calculations. The audited registry was assembled on 12 September 2026; these dates should be distinguished from each other and from the original export timestamp, which was not documented. Reporting follows the applicable principles of STROBE and RECORD for cohort definition, variable provenance and limitations of routinely collected data [8,9].

### 2.2. Intended Biological Study and Feasibility Assessment

The intended retrospective study would relate early placentation information to later foetal adaptation and tolerance of labour. Its required domains were first-trimester uterine artery pulsatility index and pregnancy-associated plasma protein A, timed later foetal assessments, maternal clinical phenotype, actual labour exposure, the indication and supporting intrapartum evidence for operative delivery, and neonatal condition. Angiogenic measurements would support an expanded model but were not required for a reduced early-marker model.

A clinically interpretable intrapartum-compromise endpoint was intended to incorporate documented labour exposure and evidence supporting suspected foetal compromise, with objective neonatal acid–base information. Suspected compromise prompting intervention, biochemical acidaemia and neonatal morbidity are related but distinct observations. Neither emergency caesarean delivery nor an isolated Apgar score was accepted as an interchangeable substitute. Professional guidance specifically cautions against using the Apgar score alone to establish asphyxia [12].

The feasibility assessment examined whether the necessary domains existed before fitting biological association models. No association model was fitted, no endpoint was reconstructed by substituting an available delivery-mode category, and no biochemical threshold or composite endpoint was retrospectively optimised. The intended analysis is described at the domain level; it is not presented as an executed or prospectively registered clinical protocol. A future biological study would need to lock its precise endpoint algorithm, thresholds and analysis plan before testing associations.

### 2.3. Main Cohort and Analytical Unit

The sole source cohort comprised 9008 technically eligible administrative keys constructed from maternal identifier, case number and foetal number. Eligibility required crown–rump length of 45–84 mm, pregnancy-associated plasma protein A of 0.01–20 multiples of the median, and uterine artery pulsatility index of 0.1–10 multiples of the median on the same first-trimester source row. These ranges were operational eligibility and plausibility filters, not disease thresholds or analysed exposure distributions.

Provenance of the eligibility limits and successive exclusions: The crown-rump length interval of 45–84 mm is the interval specified by the Foetal Medicine Foundation for first-trimester nuchal translucency assessment [13]. The multiple-of-the-median ranges reflect software-defined acceptance limits in the ASTRAIA workflow, as confirmed by the clinical investigators, rather than investigator-selected analytical thresholds; the present audit could not verify the software configuration itself. They were applied as operational eligibility rules and were not interpreted as disease thresholds, as validated biological reference ranges, or as evidence that values outside them represent pathology. Applied in the order stated to the 65,087 data rows of the source export, the marker rules excluded 47,052 rows at crown-rump length, a further 4890 at pregnancy-associated plasma protein A, and a further 3894 at uterine artery pulsatility index; the operational singleton rules then excluded 101 rows with a foetal number above 1, 103 rows with a recorded chorionicity value, and 13 rows belonging to a maternal identifier and case number in which any foetal number above 1 appeared. This left 9034 rows representing 9027 administrative keys, from which the analysis-period restriction removed 19 keys. These counts are dependent on the order in which the rules are applied and include rows with absent as well as out-of-range values; they are not counts of excluded women, pregnancies or diagnoses.

The operational singleton rule required foetal number 1 and no foetal number above 1 within the same maternal identifier and case number; an unfilled chorionicity field was used only as an additional criterion. An empty chorionicity field may represent missing information rather than a singleton pregnancy, and on its own it was not treated as establishing singleton status. This algorithm does not replace clinical confirmation of singleton status. One index examination per administrative key was retained by choosing the earliest chronological eligible examination, with source-row order resolving ties. The resulting 9008 keys represented 8090 maternal identifiers. A unique administrative key was not assumed to prove a unique biological pregnancy; repeated maternal identifiers and potential administrative duplication were retained within the defined cohort.

Of the 8090 maternal identifiers, 7212 contributed one administrative key, 844 contributed two, 31 contributed three, two contributed four and one contributed seven. Thus 878 women (10.9%) contributed more than one key, together accounting for 1796 keys (19.9% of the cohort). Keys sharing a maternal identifier were neither merged nor classified as duplicates, because the export does not establish whether they represent distinct pregnancies, repeated registration of one pregnancy, or both. The administrative key remained the unit of analysis; a sensitivity analysis restricting each woman to a single key is described in Section 2.7.

Technical-review flags: During construction of the derived registry, a key was flagged for technical review if at least one entry existed for it in the processing issue log. Entries were generated by a second eligible key for the same woman on the same index date, by more than one eligible first-trimester row, by a missing or zero index gestational age, by an index-row birth date earlier than the index examination or more than 300 days after it, or by a visit-chronology alert. Seventy-three keys in the cohort carried 74 open entries, with one key accounting for two (Supplementary Table S2). These are flags generated by the analysis pipeline, not statuses recorded in the clinical database, and they are not confirmed duplicate pregnancies. Flagged keys were retained without merging or exclusion: the flags qualify clinical interpretation rather than cohort membership.

Records were not removed from the source cohort because delivery information was absent. Thus, all full-cohort descriptions use the same denominator, and outcome-maturity analyses are explicitly nested subsets. Data-processing checks assessed date and gestational-age consistency. Retained administrative anomalies were treated as limitations on clinical interpretation rather than silently corrected or converted into additional cohort exclusions.

### 2.4. Observation Windows and Field Provenance

A second-trimester examination was counted if a record existed at 18 + 0–24 + 6 weeks, and a third-trimester examination was counted if a record existed at 28 + 0–42 + 6 weeks. Longitudinal examination availability required at least one examination in each window for the same administrative key. Multiple rows or examinations within a window contributed only one availability indicator. A source-audit sensitivity check did not change the full-cohort window counts. It required, for each later examination, a recorded gestational age of 4–45 weeks; agreement within 28 days between the gestational origin derived from that examination and the gestational origin derived from the index examination, each origin being the examination date minus the recorded gestational age in days; and an examination date between 100 days before and 280 days after the index date.

The two windows were chosen to correspond to the routine mid-trimester anomaly assessment and to the later growth-assessment interval in use at the centre, and they were separated so that an examination could contribute to only one of them. The thresholds of the source-audit sensitivity check were set wide enough to admit any plausible clinical examination while excluding records whose dating was internally inconsistent; they were intended to test the stability of the window counts, not to define eligibility.

These indicators measure the presence of examinations. They do not establish the availability, technical quality or comparability of estimated foetal weight measurements. No estimated foetal weight, centile, z-score, growth velocity or trajectory was extracted for this manuscript. First-trimester marker values were used only to reproduce the fixed eligibility definition, without analysis of their distributions or associations.

These indicators describe the state of the analysed export at the time of extraction. Because the export carries no field-level update timestamps, it records whether an item was present when the data were extracted, not when that item became available during care. These proportions describe documentation available at extraction and may change with subsequent outcome retrieval or correction. The clinical database has continued to be completed since the extraction, particularly for recent index years.

Delivery mode, birthweight availability and indication documentation were taken from the episode registry, which consolidated information within administrative keys. A single distinct nonmissing field value could be retained; contradictory values were not resolved by an arbitrary choice. Mean arterial pressure (MAP), placental growth factor (PlGF), 1 min Apgar and neonatal transfer availability were retrieved from the designated index source row, with any-visit availability reported separately where the two differ. A field appearing on that row indicates its presence in the export, not that a later outcome was already known at the time of the antenatal examination. The file contains no field-level update timestamps.

Field presence was defined as nonblank, without imputing absent values. A recorded Apgar of zero was retained as documented, and neonatal transfer entries were counted as entries rather than interpreted as an event incidence. First-trimester PlGF expressed as multiples of the median was kept separate from raw PlGF and from any-visit availability. Consequently, counts from these different fields or extraction scopes are not interchangeable. An unfilled diagnostic field was not treated as evidence of disease absence.

Record-level linkage. The derived longitudinal registry was linked to the source export by the physical source-row position of the index examination rather than by reconstructed identifiers, so that each registry episode resolved to exactly one export row. Linkage was verified by comparison of a 500-record sample, in which all 500 episodes matched their intended source row on maternal identifier, case number, foetal number and examination date. The linkage was internal to a single clinical database and its derived registry; no external data source was linked and no probabilistic matching was used.

Source-row position was used because the export provides no stable record identifier. This is a property of the data architecture rather than an analytical choice, and it is a limitation: row order was not documented as a guaranteed feature of the extraction process, so an identical linkage cannot be assumed to be reproducible from a fresh export. The derived registry is therefore versioned and retained, and the verification sample is reported above so that the correspondence can be re-examined on the same files.

### 2.5. Outcome Maturity

To limit incomplete follow-up near the analysis cutoff, we applied a gestational-age-based outcome-maturity rule. For each key with a positive recorded first-trimester gestational age, a reference date at 42 + 0 weeks was calculated as the index examination date plus (294—gestational age at index examination in days). A key was classified as outcome-mature at lag L if the reference date plus L days fell on or before 31 August 2026.

The principal lag was 90 days, with 0- and 180-day sensitivity analyses. The 42 + 0 reference is an operational late-gestation anchor, not a claim about the latest biologically possible delivery date. The lag allows additional time for routine return of information but does not establish the actual date of delivery or receipt. We avoided conditioning eligibility on a known delivery date because delivery-date availability itself depends on outcome capture.

Outcome maturity is a temporal eligibility criterion, not an ascertainment status. A key classified as mature had passed a gestational-age-derived date plus the chosen lag before the analysis cutoff; this says nothing about whether the pregnancy outcome occurred, was known, or was recorded. Only a minority of mature keys carry documented delivery information, and the term should be read accordingly throughout.

The 90-day principal lag was a pragmatic interval chosen to allow time for routine return of information after a late-gestation reference date. It was not derived from measured reporting delays, which the export does not permit us to estimate, and it was not calibrated against any external standard. For this reason, it is reported together with the 0- and 180-day analyses rather than as a validated threshold. The three lags yielded 8618, 8436 and 8254 mature keys, respectively.

Ten keys had absent or zero index gestational age and were unclassifiable; they remained in full-cohort antenatal descriptions but were excluded from each maturity-restricted analysis. Other keys not meeting the date threshold were excluded only from the relevant maturity subset. First-trimester field availability was described in the full cohort. For direct temporal comparison, later examination availability and delivery availability were also calculated together within the same principal mature subset.

Calendar epochs were 2010–2014, 2015–2019, 2020–2023 and 2024–2026, assigned by index examination year. The maturity rule was introduced after preliminary unrestricted temporal inspection identified potential incomplete follow-up in the most recent calendar years, then fixed for the final analysis together with both sensitivity lags. We therefore do not describe this rule as prospectively prespecified before data inspection. All epochs were retained; the last epoch contains only records meeting the chosen maturity threshold for outcome analyses.

### 2.6. Mechanisms of Unavailable Information and Adjudicability

We used three mechanisms to describe feasibility barriers. “Not generated” denotes absence of the clinical exposure or event needed to produce an observation, as when delivery occurs before labour. “Not measured” denotes a clinical or biological quantity that was not assessed within the relevant data-generating pathway. “Not documented” denotes information unavailable in the analysed record even though the event or assessment may have occurred. The categories describe mechanisms at the variable or pathway level and are not mutually exclusive patient groups. Where the evidence could not distinguish nonmeasurement from failure of information transfer, the mechanism was left unresolved.

Assignment to the three mechanisms followed explicit rules. A key was assigned to “not generated” only where a recorded label was compatible with absence of the relevant exposure—in practice, the elective-caesarean label for labour exposure—and such an assignment is proxy-based rather than verified. “Not measured” required positive investigator knowledge that the measurement did not form part of practice within the study data pathway, as for cord blood gases; absence of a value was never on its own sufficient. “Not documented” was assigned where a field existed in the analysed structure and was empty, or where no corresponding field existed in the analysed export. Where non-measurement could not be distinguished from failure of information to reach the record—the typical situation for a sparsely populated field about which no investigator knowledge was available, such as placental growth factor—the observation was left unresolved rather than assigned to a mechanism and is reported as unresolved.

Within the third mechanism, we further distinguish two subtypes, without introducing an additional mechanism: record-level absence, where the field exists in the analysed export but is empty, and field-level absence, where no corresponding field exists in the analysed export at all. Where another extraction from the same clinical database does contain the field, this is an omission from the analysed export rather than evidence that the field was never configured in the clinical database. The distinction is descriptive rather than taxonomic, but the two have different remedies.

For the first mechanism, absence of labour was inferred operationally from the database label “elective CS”, not established from a separate labour-onset variable. This interpretation may misclassify a planned caesarean performed after labour began. Conversely, “emergency CS” did not establish that labour occurred or that foetal compromise caused the intervention. The number of elective labels quantifies the group consistent with a non-generated labour exposure under this coding assumption; it is not a validated count of all births without labour. Recording labour onset independently of delivery mode is supported by the WHO Robson classification framework [14].

Throughout this manuscript, the elective-caesarean label is therefore described as a proxy-based classification. It is not a validated measure of absence of labour, and at the level of an individual pregnancy it cannot establish that the exposure of interest did not occur.

For cord gases, the clinical investigators confirmed that umbilical cord blood gas analysis was not part of routine postpartum practice within the study data pathway during the period examined. This confirmation, together with no recorded cord pH or base-deficit values in the supplied data, supported classifying cord gases as an investigator-confirmed structural zero rather than merely an unexported column. This classification concerns the study pathway; individual delivery records from all external institutions were not reviewed to certify that no isolated measurement occurred elsewhere.

Adjudicability was treated separately as a validity dimension. It concerns whether a recorded diagnosis or indication is supported by sufficient clinical information for reproducible classification. A populated indication is not necessarily an adjudicable foetal-compromise endpoint, and an unadjudicated label is not necessarily false. No case-level adjudication yield, diagnostic accuracy or adjudicated biological outcome is reported here.

This section addresses the availability and adjudicability of information. It does not assess validity in the sense of accuracy against an external reference, which would require a source of truth outside the analysed pathway and was not undertaken here. Validity is accordingly treated as a limitation relevant to future biological inference rather than as an outcome assessed in the present study.

### 2.7. Descriptive Analysis

We report counts and proportions with explicit denominators. Availability was summarised for the full cohort, for the principal outcome-mature subset and across the four epochs. Delivery-mode composition in the longitudinally observable subset was reported for both the full cohort and the principal mature subset. The full-cohort cascade is an inventory of recorded observations; maturity-restricted proportions are the principal estimates of delivery-data availability.

The conditional analysis used only outcome-mature keys with known delivery mode. Its numerators were documented indication and an elective-caesarean label. These proportions describe the composition and documentation of captured records, not outcomes in all eligible pregnancies. We calculated descriptive percentage-point changes between the first and last epochs and compared them under the alternative maturity lags. No hypothesis tests, imputation, risk adjustment or clinical event-rate models were performed. The administrative units and repeated maternal identifiers would also require attention to clustering in any future inferential study.

To address repeated maternal contribution, a descriptive sensitivity analysis retained the earliest eligible key for each maternal identifier, using source-row order to resolve date ties, and reapplied the same maturity rule. This retained 8090 keys, of which 7587 were outcome-mature at the principal lag. The analysis limits each woman to one key; it does not adjudicate whether separate keys represent distinct biological pregnancies.

## 3. Results

### 3.1. Cohort and Maturity Denominators

The full cohort comprised 9008 administrative keys from 8090 maternal identifiers. Epoch sizes were 1632, 2939, 2725 and 1712. At the principal 90-day lag, 8436 keys were outcome-mature and 572 were excluded from outcome-availability analyses: 562 had not met the date threshold and 10 were unclassifiable because index gestational age was absent or zero. Mature epoch denominators were 1631, 2936, 2720 and 1149. The 0- and 180-day lags yielded 8618 and 8254 mature keys, with total exclusions of 390 and 754, respectively, including the same 10 unclassifiable keys.

### 3.2. Availability of the Intended Longitudinal Sequence

In the full cohort, 6875/9008 keys (76.3%) had a second-trimester examination and 2717/9008 (30.2%) had examinations in both later windows. Among those 2717, delivery mode was known for 893 (32.9%) and absent for 1824 (67.1%). The three recorded mode categories were exhaustive: 679 elective caesareans, 162 vaginal deliveries and 52 emergency caesareans, corresponding to 76.0%, 18.1% and 5.8% of the 893 known modes. The availability cascade for the full cohort and the outcome-mature subset is illustrated in Figure 1.

Thus, 679/893 recorded deliveries in the longitudinally observable subset carried an elective-caesarean label. Under the stated coding assumption, this group was consistent with absence of the labour exposure required by the intended study. The remaining 214 vaginal or emergency-caesarean records were not equivalent to 214 validated labour-compromise observations, because emergency delivery did not verify labour onset or indication.

Expressed against the original cohort, these stages retain 76.3%, 30.2% and 9.9% of the 9008 eligible keys; expressed against the immediately preceding stage, they retain 76.3%, 39.5% and 32.9%. The presence of an examination in both windows does not establish that the measurements required for the intended biological analysis are available or technically comparable, so the 2717 keys are described as records with documented examinations in both predefined windows rather than as an available longitudinal biological dataset.

Within the principal mature subset, 6523/8436 keys (77.3%) had a second-trimester examination and 2543/8436 (30.1%) had both later examinations. Delivery mode was known in 883/2543 (34.7%), comprising 674 elective caesareans (76.3%), 159 vaginal deliveries (18.0%) and 50 emergency caesareans (5.7%); 1660/2543 (65.3%) lacked delivery mode. These results were consistent with the composition of the full longitudinal subset.

These proportions describe documentation available at extraction and may change with subsequent outcome retrieval or correction; completion has continued since the extraction, particularly for recent index years.

### 3.3. Measurement and Documentation Barriers

At the index source row, MAP was documented in 1774/9008 keys (19.7%) and PlGF multiples of the median in 23/9008 (0.26%), with the same count on any recorded visit. The broader source audit identified raw PlGF entries at any recorded visit in 33/9008 keys (0.37%). This latter figure describes a different field. The available data did not support a complete angiogenic profile; absence of those fields alone could not establish whether all corresponding tests were never measured outside the source pathway.

Cord blood gases were a structural zero across the study pathway, supported by investigator confirmation and absence of recorded values. Consequently, the intended objective neonatal acid–base component could not be reconstructed, regardless of the availability of earlier examinations or delivery labels.

The analysed source export contained a field for the 1 min Apgar score but no corresponding field for the 5 min Apgar score. Both the FIGO consensus guidance and the ACOG/American Academy of Pediatrics statement describe the 1 min score as having a comparatively weak relationship with intrapartum hypoxia and acidosis, while the 5 min score carries the stronger association with neonatal death and later neurological outcome [2,12]. The clinically more informative later assessment therefore could not be reconstructed from the analysed export, irrespective of whether it had been assessed at delivery. A separate extraction from the same clinical database does contain a 5 min Apgar field, populated in under 2% of its records; the two observations together illustrate that a variable may be unavailable either because the field is absent from the analysed export or because the existing field is largely empty.

The two extractions were produced from the same clinical database under different export configurations; the investigators report that the recorded field set is configurable, and the 5 min Apgar field was not among those exported for the dataset analysed here. It was not incorporated because the analysed export and its derived registry were frozen and versioned before analysis, and substituting a later extraction would have replaced the dataset on which every figure reported here is based. The limitation therefore concerns the analysed export rather than the clinical database as a whole.

The clinical investigators also confirmed that historical collection of neonatal outcome information was selective. When patients reported a 1 min Apgar score of 8–10, the 5 min score was generally not requested during follow-up. Conversely, a lower initial score or reported neonatal concerns prompted additional questions about the 5 min Apgar score and subsequent neonatal condition. This practice concerned retrospective information retrieval rather than neonatal assessment at delivery. It may partly explain the sparse documentation of the 5 min Apgar score observed in other extractions from the same clinical database. Its contribution cannot be quantified from the analysed dataset.

In the principal mature subset, delivery mode was documented in 2387/8436 keys (28.3%), birthweight in 2409 (28.6%), delivery indication in 188 (2.2%), 1 min Apgar in 1523 (18.1%), and a neonatal transfer entry in 5 (0.06%). The full-cohort indication count was 191/9008 (2.1%). These are documentation counts; in particular, the low number of transfer entries cannot be interpreted as a low neonatal morbidity rate. Table 1 relates the observed availability to the requirements of the intended study.

### 3.4. Divergent Temporal Patterns

Within the same mature denominators, longitudinal examination availability increased from 431/1631 (26.4%) in 2010–2014 to 514/1149 (44.7%) in 2024–2026, a rise of 18.3 percentage points. The intermediate proportions were 22.0% and 35.0%, so the pattern was not a monotonic increase across all four epochs. Second-trimester examination availability alone was 76.7%, 78.0%, 78.9% and 72.6%. The divergent patterns of antenatal examination availability and delivery-data capture across calendar epochs are illustrated in Figure 2.

Delivery-mode capture declined across the four epochs from 932/1631 (57.1%) to 928/2936 (31.6%), 404/2720 (14.9%) and 123/1149 (10.7%). The first-to-last difference was −46.4 percentage points, with the latest proportion approximately one fifth of the earliest. Birthweight availability followed a similar pattern, from 56.8% to 11.7%. Delivery-indication documentation remained uncommon but increased from 1.0% to 4.5% of all mature keys.

This decline was not solely a denominator effect. The absolute numbers of outcome-mature records with a documented delivery mode were 932, 928, 404 and 123 across the four epochs (approximately 186, 186, 101 and 46 records per calendar year, although the epochs are of unequal length and the last is maturity-restricted, so the annual figures are descriptive only). Both the proportion and the absolute capture of delivery information therefore declined.

Two features of the data limit how this contrast may be read. First, the epochs are of unequal duration and the 2024–2026 epoch is additionally restricted by the maturity rule and truncated by the analysis cutoff, so these are differences in availability between calendar-period-specific subsets rather than a continuous temporal trend. Second, and more consequentially, the routes by which outcome information returned to the record—completion at a subsequent pregnancy, reminders, and telephone contact prompted by incomplete or inconsistent entries—all accumulate with the time a record has spent in the database. A key indexed in 2010 has had sixteen years of such opportunities; one indexed in 2024 has had two. The maturity rule guarantees a minimum interval after a late-gestation reference date, but it does not equalise the opportunity for retrieval across index years. Part of the observed gradient may therefore reflect cumulative recovery in older records rather than a change in contemporaneous reporting, and the present data cannot separate the two.

Outcome information did not decline uniformly. One-minute Apgar documentation fell from 837/1631 (51.3%) to 73/1149 (6.4%), more steeply than delivery-mode capture, and was lowest at 120/2720 (4.4%) in 2020–2023, when delivery mode was still available in 14.9% of mature records. Declining outcome capture was therefore accompanied by differential loss of detail within the delivery information that did return.

First-trimester MAP documentation, evaluated in the full cohort, was 1/1632 (0.06%), 2/2939 (0.07%), 141/2725 (5.2%) and 1630/1712 (95.2%). This marked change contrasted with declining delivery-data capture. Recorded PlGF multiples of the median remained uncommon, at 0, 0, 3 and 20 keys across the epochs. Table 2 presents the exact denominators and counts. The aggregate contrast shows improved capture of a specific antenatal measurement alongside poorer delivery documentation; it does not identify the cause of either change. We are unable to determine whether the rise in MAP availability reflects a change in clinical measurement, in recording practice, in database structure or mandatory-field configuration, or in the extraction itself; no corresponding change is documented in the available material. The finding therefore describes availability of the MAP field in the analysed data and does not by itself demonstrate a corresponding increase in measurement in clinical practice.

### 3.5. Changes in the Composition and Informational Content of Returned Outcomes

Among the 2387 mature keys with known delivery mode, the proportion with a documented indication increased from 16/932 (1.7%) in the first epoch to 25/928 (2.7%), 95/404 (23.5%) and 52/123 (42.3%). The aggregate proportion was 188/2387 (7.9%). Thus, the latest known-mode records more often included an indication despite the reduction in the proportion of all mature records with a known mode. The last-epoch proportion rests on 123 records and should be read with that denominator in view.

Elective-caesarean labels accounted for 756/932 (81.1%), 757/928 (81.6%), 277/404 (68.6%) and 77/123 (62.6%) of known modes, with an overall proportion of 1867/2387 (78.2%). This change describes the captured subset. It does not establish a change in the true elective-caesarean rate, a rise in foetal compromise, or improved validity of the recorded indications.

The denominator of this analysis is restricted to mature keys with a documented delivery mode, and that subset itself contracts across the epochs, from 932 to 928, 404 and 123 records. The rise in documented indications is therefore a statement about the composition of the records that returned, and it is compatible with a change in which records return rather than with improved documentation of deliveries in general.

These observations should not be summarised as a general improvement in documentation quality, because they run in opposite directions for different fields: among the outcomes that returned, structured indication text became more frequent while 1 min Apgar documentation became less frequent. What changed was the composition and informational content of returned outcomes, not their quality along a single axis.

### 3.6. Recorded Labels and the Information Needed to Reproduce a Phenotype

Recorded diagnostic labels also varied in the amount of supporting information available. Pre-eclampsia was flagged in 39 eligible records in the principal field and in nine records in a separate pre-eclampsia field that could not be combined without a definitive data dictionary; the two fields did not co-occur within the same key. Gestational week of onset was available for 28 recorded episodes. These observations were not used to adjudicate or refute individual diagnoses; rather, they illustrate the distinction between the presence of a diagnostic label and the information required to reproduce a research phenotype retrospectively.

The maternal outcome domain illustrates the same distinctions. The analysed export carries an extensive maternal history at booking—chronic hypertension, diabetes, renal, cardiac, hepatic, thyroid and autoimmune disease, coagulopathy, thrombophilia, systemic lupus erythematosus, antiphospholipid syndrome, epilepsy, previous pre-eclampsia and previous low-birthweight infant, among others—but only two fields recording a maternal outcome of the index pregnancy, both for pre-eclampsia. No field in the analysed export records eclampsia, HELLP syndrome, postpartum haemorrhage, maternal critical care admission, maternal death or gestational diabetes as an outcome. The clinical investigators report that the software permits additional and configurable fields, so this pattern reflects what the service chose to capture rather than a limit of the application: hypertensive disease was the focus of clinical and research interest, gestational diabetes was not routinely screened for by the clinicians providing the women’s general antenatal care during much of the period, and the centre neither performed nor received such testing, and severe maternal events would have been recorded had they become known to the centre. These are statements supplied by the clinical investigators about prevailing practice; they were not verified for individual pregnancies in the analysed records, which contain no field in which such verification could be recorded. Their absence from the export therefore cannot distinguish events that did not occur from events that were never reported back.

### 3.7. Sensitivity to Outcome Maturity

At lag 0, the last epoch included 1331 mature keys, of which 606 (45.5%) had both later examinations and 132 (9.9%) had known delivery mode. At lag 180, the corresponding denominator was 967, with 421 (43.5%) and 106 (11.0%), respectively. The first three epoch denominators and their registry-based numerators were unchanged across the three lags. The rise in longitudinal examination availability and decline in delivery capture therefore persisted under both alternative rules.

The conditional last-epoch indication proportions were 54/132 (40.9%) at lag 0 and 47/106 (44.3%) at lag 180, compared with 52/123 (42.3%) in the principal analysis. Elective-caesarean proportions in those known-mode subsets were 62.1%, 63.2% and 62.6%, respectively. These sensitivity results reduce concern that the headline contrast is solely due to records too recent to have reached delivery, while leaving uncertainty about actual information-return delays and selective capture.

In the sensitivity analysis retaining the earliest eligible key per woman, 8090 keys remained, of which 7587 were outcome-mature. The divergence persisted: availability of examinations in both later windows rose from 406/1560 (26.0%) in 2010–2014 to 462/1047 (44.1%) in 2024–2026, while delivery-mode documentation fell from 879/1560 (56.3%) to 101/1047 (9.6%). Restricting each woman to a single key therefore does not account for the contrast.

## 4. Discussion

### 4.1. Principal Findings

The main finding was a divergence between longitudinal antenatal observation and delivery-data capture within one consistently defined source cohort. Between the first and last epochs, availability of examinations in both later windows rose by 18.3 percentage points while delivery-mode capture fell by 46.4. What matters for feasibility is the direction rather than the size of these movements: the part of the record generated inside the service became denser, while the part generated elsewhere became sparser, so the two ends of the intended sequence drifted apart rather than together.

The divergence cannot be explained solely by expansion of the antenatal denominator: the absolute number of records with a documented delivery mode also fell substantially, from 932 in the first epoch to 123 in the last.

The intended biological sequence could be partially traced through antenatal visits, but it could not be completed through a valid intrapartum endpoint. Many known-mode records carried an elective-caesarean label consistent with no labour exposure, while labour onset was not independently verified. Cord gases were structurally unavailable within the study pathway, no 5 min Apgar field existed in the analysed export, and indications were documented for only a small fraction of mature records. This establishes a limitation of the available data for the intended question, not evidence for or against reduced foetal reserve.

### 4.2. Why the Three Mechanisms Require Different Responses

The distinction between not generated, not measured and not documented changes the remedy. An intrapartum response cannot be recovered by filling an empty cell if labour did not occur. If labour occurred but the intended acid–base measurement was not undertaken, retrospective data cleaning cannot recreate the specimen. If information exists in a delivery institution but has not reached the antenatal record, structured transfer or authorised retrieval may recover it. The same key may encounter more than one of these barriers.

Documentation failure can occur either within a recorded field or through the absence of the field itself. Where a field exists and is empty, the remedy is completion: prompts, mandatory entry, feedback on completeness. Where the analysed export contains no corresponding field, completion at the point of care cannot produce the variable. The 1 min and 5 min Apgar scores illustrate the two situations within a single clinical measure. These two absences are moreover not independent of the missing acid–base information: professional guidance directs that an umbilical artery blood gas be obtained from a clamped segment of cord when the 5 min Apgar score is 5 or less [12]. The score absent from the analysed export is therefore also the score that triggers the measurement whose absence is reported here, so one decision about which fields to record can remove both an outcome variable and the documented prompt for the measurement that would have supported it. It does not establish that the 5 min assessment was not performed, nor that the absence of one field caused the absence of the other. For service planning these are different projects with different owners, and conflating them under a single heading of missing data obscures which one is required.

The remedy for an absent field should also be described accurately. The clinical investigators report that the application supports configurable fields, so the remedy is in most cases extension and configuration of the recorded field set rather than redesign of the underlying application. The practical distinction we draw is therefore between completing fields that exist and deciding which fields should exist. The second is a governance and configuration decision, not a technical obstacle, and that makes it a cheaper remedy than our framing might otherwise suggest—and a decision for which a service is answerable.

The 5 min Apgar example illustrates a further distinction between the existence of a clinical measurement and its selective retrieval into an antenatal record. Although the score is routinely assessed in delivery care, its retrospective collection by the study centre was influenced by the initially reported neonatal condition. A reassuring 1 min score generally did not prompt additional questioning, whereas less reassuring findings led to more detailed follow-up. Consequently, records containing a 5 min Apgar score may represent a selected subset rather than a representative sample of neonatal outcomes. This investigator-described retrieval practice provides a plausible explanation for incomplete documentation but does not establish the magnitude or direction of selection bias in the analysed cohort.

These mechanisms should not be read as a ranking of care quality. Elective delivery may be clinically indicated, and this analysis did not assess the appropriateness of caesarean decisions. The database label also remains an imperfect indicator of actual labour exposure. The WHO Robson system demonstrates the value of keeping labour onset distinct from delivery mode, but it is a classification of obstetric groups rather than an adjudication of operative indication [14]. The same manual addresses the architectural case directly: where labour onset cannot be classified because the information was not collected, it recommends adding a dedicated field to the admission record so that every woman is assigned one of spontaneous labour, induced labour or pre-labour caesarean, and it treats the size of the resulting unclassifiable group as an indicator of the quality of a hospital’s data rather than as a residual to be omitted [14].

The structural-zero designation for cord gases is stronger than an inference from an empty field because it incorporates investigator knowledge of measurement practice. Its scope remains the study pathway. A birth elsewhere could have generated records not represented here. Accordingly, sparse angiogenic or neonatal-transfer fields were not automatically given the same structural interpretation. Differentiating evidence of nonmeasurement from absence of documentation avoids overstating what an export can reveal.

The maternal outcome domain shows all three mechanisms at once. Gestational diabetes was largely not screened for by the clinicians providing general antenatal care during much of the study period, so the relevant measurements were not available within the study pathway; whether testing occurred elsewhere could not be established from the analysed records. The investigators’ account of prevailing practice makes non-measurement the likely explanation, but this is weaker evidence than the investigator-supplied, pathway-level confirmation available for cord gases, because it concerns care delivered outside the centre rather than practice within the study pathway. Adding a field would not recover an observation that was never made, and the remedy in that case is a change in clinical measurement, which the centre reports it now intends to ask about. The case also marks the limit of patient-reported outcome recovery, which was one of the principal return routes available to the centre: where testing did not occur, the information cannot be retrieved by asking the woman, because she has nothing to report. A measurement that was never made is unavailable to every retrieval route, not only to the record. Pre-eclampsia had fields that were used, and their sparse population is an absence at record level. Severe maternal morbidity had no field and no return pathway, and here the two candidate explanations—that such events did not occur in this cohort and that they occurred but were never reported back—cannot be separated by the available data. This is the unresolved category defined in Section 2.6, applied to a domain in which the distinction matters clinically.

### 4.3. Acid–Base Evidence and the Meaning of Intrapartum Compromise

External literature supports retaining separate definitions for suspected intrapartum compromise, biochemical acidaemia and clinical neonatal morbidity. Cord pH is associated with important adverse neonatal outcomes without explaining them on its own: in a cohort of 51,519 validated arterial samples, more than 75% of neonates with the neurological outcomes studied had pH values above 7.10 [15]. A systematic review of 51 studies and 481,753 infants found strong associations between low arterial cord pH and adverse outcomes—odds ratios of 16.9 for neonatal mortality and 13.8 for hypoxic–ischaemic encephalopathy—while documenting substantial heterogeneity in pH thresholds, outcome definitions and study quality [16]. A 2023 systematic review restricted to term births in high-income countries concluded that the associations between cord blood acid–base status at delivery and long-term neurodevelopmental outcomes remain of low certainty [20], a further reason to prespecify such an endpoint rather than assume its validity. This evidence is cited to explain why the unavailable acid–base information matters for the intended future study. It is not offered as validation of the endpoint, which the present analysis did not evaluate.

Current ACOG and NICE guidance addresses foetal heart-rate interpretation and management within intrapartum assessment [3,4]; a delivery-mode label is not an equivalent indicator of hypoxic injury. The ACOG/American Academy of Pediatrics statement on Apgar likewise rejects its isolated use to diagnose asphyxia [12]. Therefore, substituting emergency caesarean or 1 min Apgar for the intended acid–base-supported endpoint would change the estimand. The endpoint should be prespecified before any future biological analysis rather than relaxed to fit the fields that happen to be available, and it cannot be relabelled as observed in this cohort.

Measurement-dependent endpoint definitions also have practical limits when transferred between settings. A definition requiring cord gases can be applied only where the requisite samples and documentation exist. This is a limitation on implementation and ascertainment, not a reason to relax physiological definitions after examining available data.

### 4.4. Care Pathways and Selective Outcome Capture

The contrast with MAP is informative because it shows that near-universal documentation of a specific antenatal field was achievable in the latest epoch. It is compatible with a change in routine antenatal recording or measurement practice. However, this observational contrast is not a natural experiment: the analysis did not establish an intervention date, a control series or a causal effect of a workflow change.

Antenatal care distributed across a different institution from delivery offers a plausible explanation for the declining return of outcome information. The conditional analysis is also compatible with selective ascertainment: fewer outcomes were captured, but the captured subset more often included an indication and less often carried an elective label. Other explanations include changes in patient mix, referral patterns, institutional interfaces, reporting behaviour or coding conventions. We did not measure these mechanisms, and the conditional data cannot determine which explanation predominated.

The fall in the elective-caesarean share of documented deliveries, from 81.1% to 62.6%, admits at least two readings that the present data cannot separate. The eligible population may have broadened: the proportion of first-trimester examinations meeting marker eligibility was 53.2% in the first epoch and 63.8% in the last, with a minimum of 48.2% in 2015–2019 (Supplementary Table S1), so the later cohorts are drawn from a wider slice of attendances. Alternatively, the subset whose outcome returned may have changed, with non-elective deliveries becoming relatively more likely to be reported back. Both are accounts of ascertainment rather than of obstetric practice, and both are consistent with the principal finding.

These explanations are generated by the observed data; none of them has been tested here. The analysis measured neither referral patterns, nor patient mix, nor institutional interfaces, nor reporting behaviour, and it cannot determine which, if any, predominated. In particular, the plausibility of fragmented antenatal-to-delivery care must not be allowed to become an inferred cause of the temporal decline. The cumulative-opportunity effect described in Section 3.4 is a further candidate that applies to every epoch simultaneously and that the present data cannot separate from the others.

National context and what it does not license: In Romania, approximately 89% of recorded births occur in public hospitals, and the national caesarean rate in 2020 was 52.9% (49.7% in public and 79.8% in private units) [21]. This context explains why an intended endpoint requiring labour is difficult to populate in this setting. It does not, however, provide a benchmark for the present cohort. Because women obtaining private foetal-medicine assessment cut across both delivery sectors, the 76.0% elective-caesarean share among documented deliveries here cannot be compared with either national figure and should not be interpreted as the practice of any institution.

For service evaluation, the practical implication is to monitor both the fraction of eligible episodes with a returned outcome and the information contained in those returned records. Improving the second measure alone can conceal deterioration in coverage. Complete-case biological analyses would additionally be conditional on return of information, which may depend on clinical course and care location; adjustment would require variables and assumptions beyond those evaluated here.

The extent of the gap became apparent to the service itself only when research questions were put to the database. Routine care had continued without it being visible, because routine care does not require the outcome to return; auditing a prediction does. That sequence is the argument of this paper reproduced at the scale of one service, and it suggests that the absence of a closed information loop tends to remain unnoticed until something other than care is asked of the record.

### 4.5. Closing the Audit Loop in Prenatal Screening

The implications extend beyond retrospective research. A prenatal screening service requires outcome ascertainment to audit its own performance. Without systematic return of delivery and maternal outcome information, a centre cannot reliably estimate screen-positive yield against subsequent disease, evaluate local calibration, investigate false-positive and false-negative pathways, or determine whether operational referral thresholds perform as intended in the population actually being screened. This requirement is not new: more than twenty years ago, an audit of prenatal screening uptake and outcome across four maternity units was made possible only by linking screening, obstetric and diagnostic records, and its authors concluded that such audit depends on maternity datasets that include the relevant outcome information [5].

The contrast documented here makes this concrete. Over the same period in which availability of a first-trimester measurement made within the service reached 95.2%, for reasons this analysis could not determine, the proportion of records with a documented delivery mode fell to approximately one in nine. Increasing sophistication of antenatal risk calculation therefore does not by itself create a learning screening system. The information loop must extend from risk estimation to verified outcome and back to the screening service.

For a fragmented care pathway, the intervention suggested by these findings is not necessarily additional antenatal measurement but structured outcome return: a minimum delivery dataset, interoperable transfer where possible, and defined responsibility for closing the episode. This is a service-design question rather than a research-methods question.

These are statements about one antenatal service. The present data show that a service in this position may be unable to evaluate its own downstream outcomes when outcome information returns incompletely. They do not establish that prenatal screening programmes generally operate as open-loop systems, nor that discontinuity of this degree exists elsewhere. The general claim we make is conditional rather than empirical: wherever prediction and outcome are separated institutionally, structured outcome ascertainment is a precondition for evaluating the prediction, and whether that condition is met is an empirical question for each setting.

Proposed improvements, grounded in the gaps documented here: On the evidence of this audit, we propose extending the existing follow-up pathway with a standard minimum outcome form, reminders anchored to an expected date of pregnancy completion, and a defined responsibility for checking returned information and closing each episode; storing the birth institution, the source of each outcome, the date of receipt and the date of entry as separate fields; distinguishing unknown, not performed and not returned as explicit states; and recording actual labour onset and exposure independently of delivery mode. These are proposals at the level of information capture and recovery, not changes to clinical care, and their effects have not been evaluated. We report them because each corresponds to a specific absence documented above, not as interventions shown to improve continuity.

The applicability of these proposals beyond the present service will depend on local care pathways, database architecture and data-sharing arrangements. We distinguish the methodological principle, which we believe is general, from the empirical findings of this single-centre study, which are not.

Three positions on the same spectrum: It is useful to describe prenatal services by the completeness of their information loop rather than by the sophistication of their prediction. Under an open information loop, screening produces a risk estimate and care continues outside the information system. Under selective outcome return, outcome information comes back for part of the cohort, as documented here. Under systematic outcome feedback, outcome linkage feeds audit and recalibration back into screening—the position occupied by national programmes that undertake active multi-source ascertainment for exactly this purpose [7]. These positions characterise systems rather than individual centres: a service cannot move along this spectrum by internal effort alone, because linkage to birth outcomes requires infrastructure, governance and a legal basis that operate above the level of any single provider. Figure 3 presents this framework.

### 4.6. Standardisation and a Minimum Dataset for Replication

For external replication, we propose a research-specific minimum dataset rather than a new consensus standard. Because it is a proposal addressed to future studies rather than a result of the present audit, and to keep this section proportionate, the dataset and its relation to existing international initiatives are set out in Supplementary Table S3 and the accompanying note. In outline, it requires a verified pregnancy identifier linked to the mother, dating and index gestational age, first-trimester marker acquisition and assay metadata, two timed later examinations, supported maternal phenotype, actual labour onset and exposure, delivery timing and mode, a structured indication with its supporting evidence, paired cord-gas sampling information where the endpoint requires it, and defined neonatal outcomes with their assessment times including the 5 min Apgar score. It is an author-derived recommendation, not a validated standard.

### 4.7. Validity and Limitations

This study has several limitations. The cohort is selected by attendance at a private antenatal centre and by technical eligibility requiring particular first-trimester fields. It therefore cannot estimate the prevalence of missing measurements or delivery outcomes among all pregnant people in Romania. Its denominator is administrative keys, not a fully adjudicated count of distinct pregnancies, and repeated maternal identifiers and retained administrative anomalies limit clinical and statistical interpretation.

Cohort entry itself depended on availability of both first-trimester markers, and marker availability changed over calendar time. Among singleton first-trimester examinations with a crown–rump length of 45–84 mm and an examination date within the analysis period, the proportion meeting full marker eligibility was 1632/3069 (53.2%), 2939/6099 (48.2%), 2725/4896 (55.7%) and 1712/2685 (63.8%) across the four epochs (Supplementary Table S1). The composition of eligible pregnancies may therefore have changed between epochs, and we cannot assume that temporal changes in documentation arose within an otherwise stable patient population. Reporting this drift is required rather than discretionary: RECORD asks authors to discuss the implications of using data that were not created to answer the research question, naming changing eligibility over time among the issues to be addressed [8].

The maturity rule deliberately used an operational late-gestation anchor rather than the actual delivery date, because delivery-date availability was itself part of the ascertainment problem. This conservative choice can exclude recent pregnancies that had already delivered, particularly preterm pregnancies. Because complicated or early deliveries may be more or less likely to return to the antenatal record, the rule may alter the composition of the most recent mature subset rather than merely reduce right censoring. The 0-, 90- and 180-day analyses show that the headline contrast does not depend on the chosen lag, but they do not resolve this selection.

Availability was defined conservatively as a documented field or examination. This does not establish measurement validity, diagnostic accuracy, completeness of a clinical encounter or compatibility of foetal assessments across time. MAP and PlGF at the designated index row used a narrower extraction scope than some any-visit audit counts, and the 5 min Apgar observation concerns the structure of the analysed export rather than the clinical database as a whole. Outcome fields were not uniformly sourced: registry-consolidated fields and index-row fields may have different sensitivities for documentation. These scopes were kept constant across epochs, but an unmeasured change in recording practice could still affect temporal comparisons.

The 90-day return lag was pragmatic and was adopted after preliminary inspection identified incomplete follow-up. It was not calibrated to observed reporting delays. The 0- and 180-day analyses show that the main contrast does not depend on that single choice, but they do not remove all delayed ascertainment or selection bias. Outcome maturity also does not identify the actual pregnancy endpoint: miscarriage, stillbirth, early delivery and continuing pregnancy were not comprehensively adjudicated. No unrecorded outcome was assigned a favourable clinical status.

Elective-caesarean labels were used as an explicitly stated proxy for absence of labour. Without independent onset information, the exact number of non-generated labour exposures is uncertain. The cord-gas structural zero depends on investigator confirmation of the study pathway, with no exhaustive external-hospital verification. No diagnosis or operative indication was subjected to the case-level adjudication needed for biological inference, and increased indication documentation cannot be described as proven improvement in accuracy.

Finally, the epoch comparisons were descriptive, used unequal calendar intervals and did not adjust for changes in patient characteristics, staffing or referral pathways. Neither the divergence in capture nor the changing composition of known outcomes establishes causation. The principal strength is transparent alignment of the intended question with one reproducible denominator, explicit maturity restrictions, and field-specific documentation rules. This makes the limits of interpretation visible before a biological model is attempted.

Three further limitations follow from the provenance of the outcome data. First, availability was measured in a single export at one moment; these proportions describe documentation available at extraction and may change with subsequent outcome retrieval or correction. Second, the routes by which outcomes returned accumulate with the time a record has spent in the database, so opportunity for retrieval is not equal across index years and part of the observed gradient may be cumulative recovery rather than contemporaneous reporting; the export carries no receipt or entry timestamps with which to separate these. Third, 73 keys carried open technical-review flags, which were retained rather than reconciled; these qualify clinical interpretation of individual records without altering any denominator.

## 5. Conclusions

In a cohort of 9008 technically eligible obstetric administrative keys, increasing longitudinal antenatal observation coexisted with declining delivery-data capture, in both relative and absolute terms and after gestational-age-based maturity restrictions. Among the outcomes that did return, the composition and informational content changed rather than improving along a single axis: structured indication text became more frequent while 1 min Apgar documentation became less frequent. An elective-caesarean label predominated in the longitudinal subset with known mode, cord gases were an investigator-confirmed structural zero within the study pathway, and no 5 min Apgar field existed in the analysed export.

At extraction, therefore, the antenatal record was denser for recent index years while delivery information was less often present for them. We describe this as a difference in documentation availability across index-year cohorts at the time of extraction, not as demonstrated deterioration in contemporaneous reporting. The intended intrapartum endpoint could not be reconstructed or tested using the available data; this is a statement about the data, not about the biological hypothesis, which remains untested. Future studies require verified labour exposure, specified measurements and reliable transfer of delivery information, with clinical adjudicability assessed separately from field availability, and with documentation gaps distinguished according to whether the field is empty or absent by design.

Meaningful evaluation of prenatal prediction requires adequate ascertainment of the relevant pregnancy outcomes, and whether that condition holds is an empirical question for each service; continuity problems of the kind documented here should be investigated in other settings rather than assumed to be general. Record linkage can recover information generated elsewhere, but it cannot recover a physiological exposure that never occurred or a measurement that was never made. Building learning prenatal systems therefore requires knowing which of these problems one is trying to solve.

## Figures and Tables

**Figure 1:**
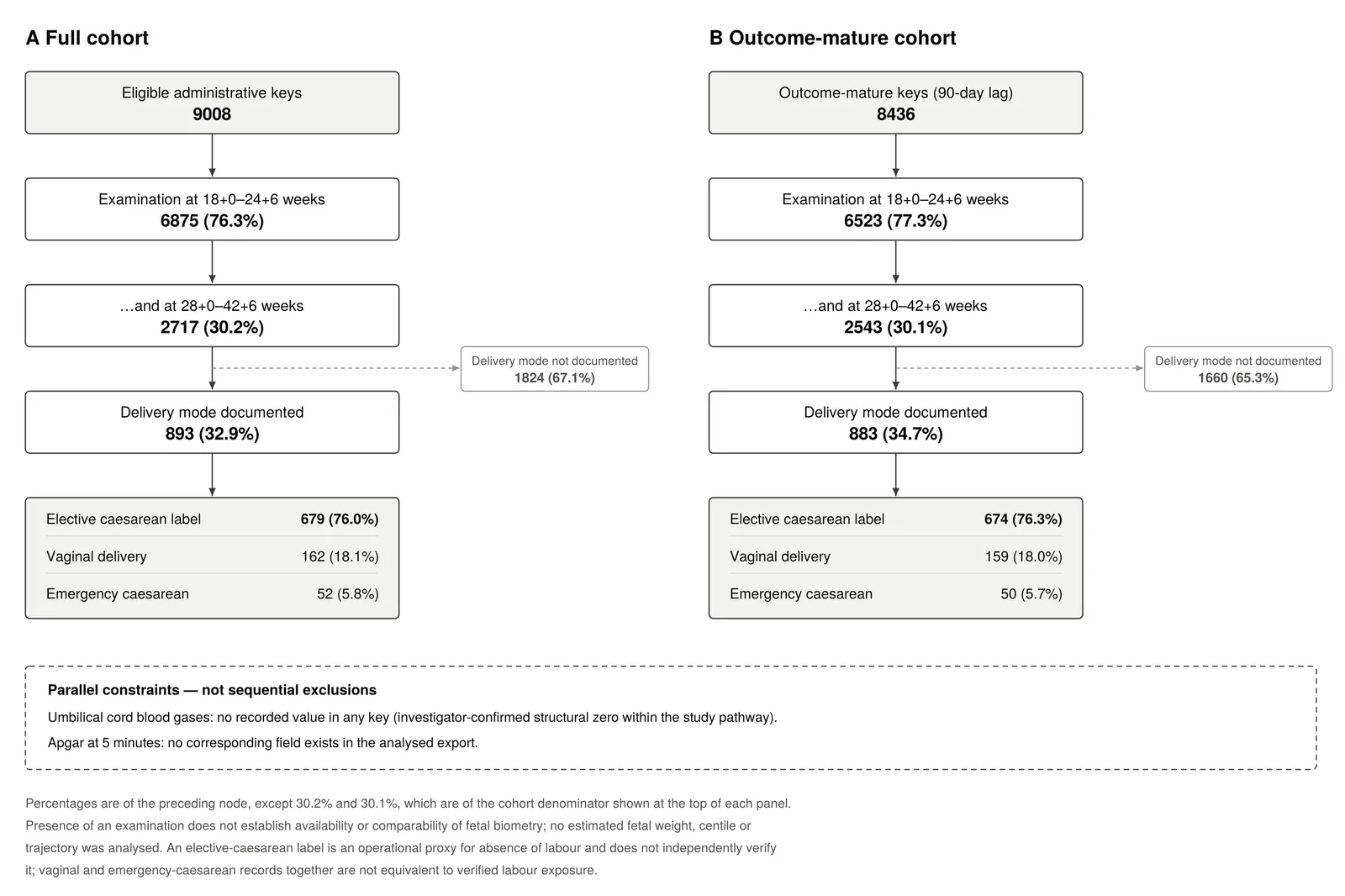
Availability of the intended longitudinal sequence. Flow of recorded observations in the full administrative cohort (**A**) and the principal outcome-mature subset (**B**). The full-cohort sequence is 9008 eligible keys, 6875 with a second-trimester examination, and 2717 with examinations in both later windows; among the latter, delivery mode is documented for 893 and not documented for 1824. Documented modes comprise 679 elective caesareans, 162 vaginal deliveries and 52 emergency caesareans. The mature branch begins with 8436 keys and shows 6523 second-trimester examinations, 2543 with both windows and 883 documented modes (674 elective, 159 vaginal, 50 emergency). Cord blood gases and the absent 5 min Apgar field are shown as parallel constraints, not as additional sequential exclusions. Percentages are of the preceding node except for the nodes showing examinations in both later windows, which are expressed as fractions of the cohort entering each panel (2717/9008, 30.2%; 2543/8436, 30.1%); relative to the preceding node these are 2717/6875 (39.5%) and 2543/6523 (39.0%). The presence of an examination does not establish the availability or comparability of foetal biometry, and an elective-caesarean label does not independently verify absence of labour; vaginal and emergency-caesarean records together do not constitute confirmed labour exposure.

**Figure 2:**
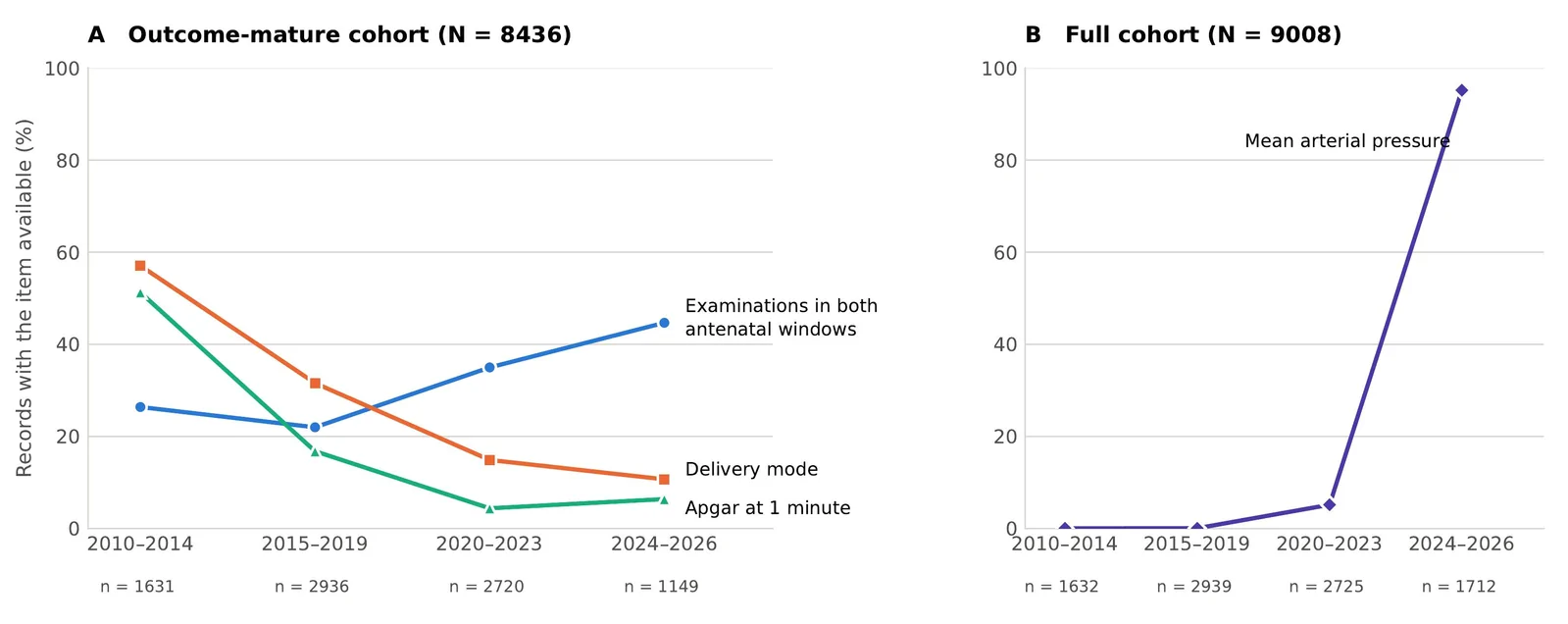
Antenatal observation and delivery capture across epochs. Panel (**A**) shows, in the principal outcome-mature subset, the availability of examinations in both antenatal windows (26.4%, 22.0%, 35.0% and 44.7%), delivery-mode capture (57.1%, 31.6%, 14.9% and 10.7%) and 1 min Apgar documentation (51.3%, 16.8%, 4.4% and 6.4%) across the four epochs. Panel (**B**) shows index-row mean arterial pressure availability in the full cohort (0.06%, 0.07%, 5.17% and 95.2%). Denominators are printed below each epoch; Panel (**B**) uses a different population scope from Panel (**A**). The panels describe documentation and do not estimate causal effects of changes in practice.

**Figure 3:**
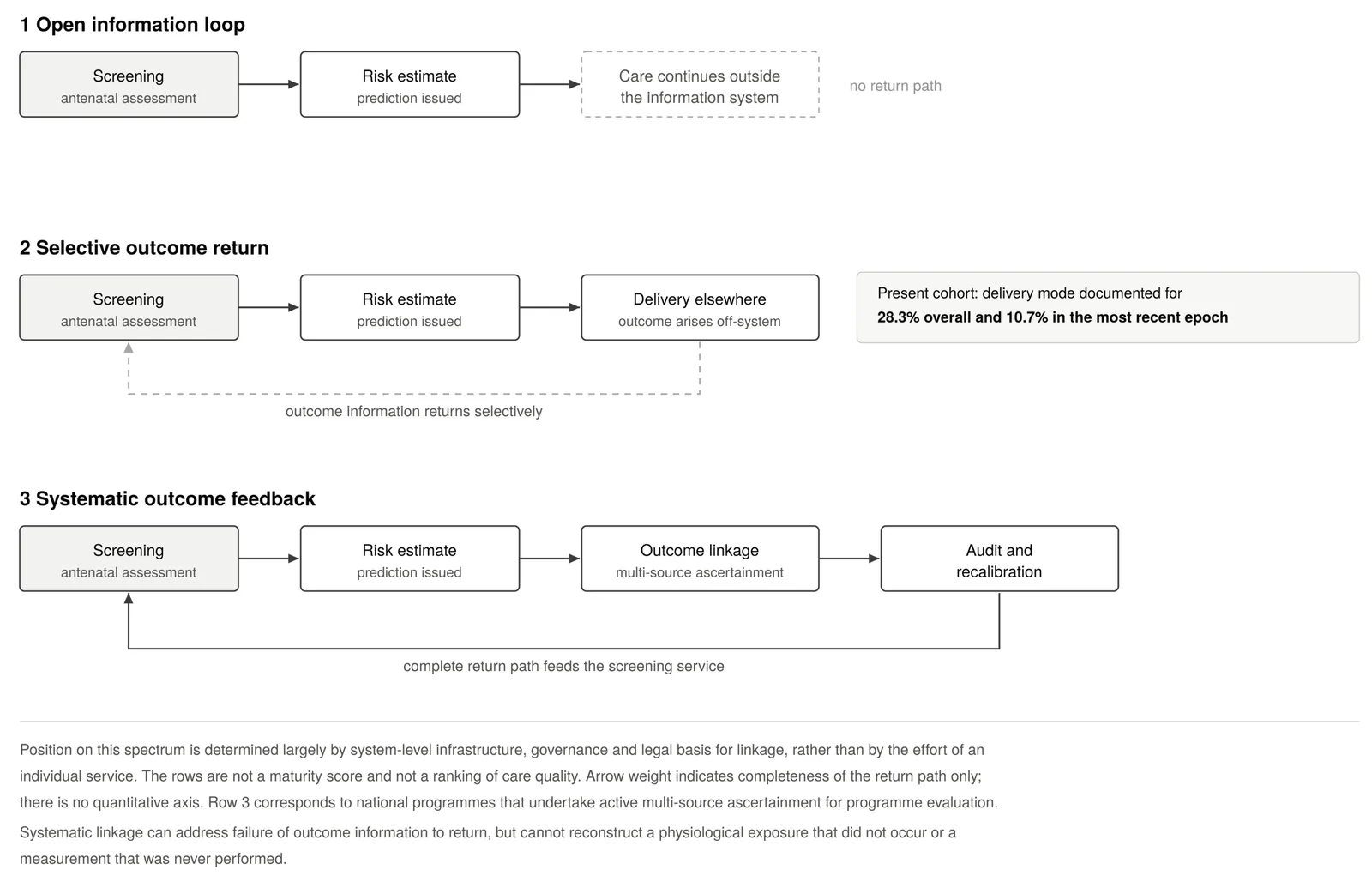
Three positions on the information loop of a prenatal service. Under an open information loop, screening produces a risk estimate and care continues outside the information system, with no return path. Under selective outcome return, outcome information comes back for part of the cohort, as in the present cohort, where delivery mode was documented for 28.3% of outcome-mature records overall and 10.7% in the most recent epoch. Under systematic outcome feedback, multi-source outcome ascertainment feeds audit and recalibration back into the screening service, the position occupied by national programmes that undertake active ascertainment for programme evaluation [7]. Position on this spectrum is determined largely by system-level infrastructure, governance and legal basis for linkage rather than by the effort of an individual service; the rows are not a maturity score and not a ranking of care quality. Arrow weight indicates completeness of the return path only, and there is no quantitative axis. Systematic linkage can address failure of outcome information to return but cannot reconstruct a physiological exposure that did not occur or a measurement that was never performed.

**Table 1:** Requirements of the intended study and observed availability.

Study Domain	Required Observation and Rationale	Available Evidence	Barrier or Qualification	Consequence
Early placentation	Eligible index examination and first-trimester marker fields; hypothesis source [1].	9008 eligible keys; marker values define eligibility only.	No marker association analysed.	Eligibility does not validate marker biology or establish representativeness.
Later foetal observation	Examinations in both defined gestational windows; usable foetal assessments needed for a biological model.	2717/9008 (30.2%); mature subset 2543/8436 (30.1%).	Examination presence only.	No foetal-weight availability, values or trajectories inferred.
Angiogenic information	Time-stamped assays and assay metadata for an expanded model.	PlGF MoM at index row 23/9008 (0.26%); raw PlGF at any visit 33/9008 (0.37%).	Not measured versus not documented unresolved; sFlt-1 unavailable.	Expanded profile cannot be reconstructed; field scopes differ.
Labour exposure	Actual onset and exposure, independently of delivery mode [2,3,4,14].	679/893 (76.0%) known modes in the full longitudinal subset were elective CS; mature 674/883 (76.3%).	Not generated, inferred from elective label; onset unverified.	No validated count of all labour-exposed records; emergency CS is not proof of labour.
Objective neonatal acid–base component	Cord pH and base deficit with vessel, sampling and validation information [15,16].	0/9008; 0/8436 mature keys.	Not measured: investigator-confirmed structural zero in study pathway.	Required acid–base component unavailable; external hospitals not exhaustively reviewed.
Delivery context	Mode, clinical indication and supporting intrapartum record [3,4,14].	Mature keys: mode 2387/8436 (28.3%); indication 188/8436 (2.2%).	Not documented—record-level absence: field present, largely empty.	Presence of an indication does not establish an adjudicable compromise endpoint.
Neonatal condition, 1 min Apgar	Standardised neonatal assessment [12,17,18,19].	Mature keys: 1523/8436 (18.1%); birthweight 2409/8436 (28.6%); transfer entry 5/8436 (0.06%).	Not documented—record-level absence.	No neonatal incidence estimated; transfer absence is not event absence.
Neonatal condition, 5 min Apgar	Clinically more informative later assessment [2,12].	No corresponding field in the analysed export; the field is present, but sparsely populated, in another extraction from the same clinical database.	Export-level omission; selective historical retrieval may also contribute to incomplete documentation.	Not reconstructible from the frozen analytical export; availability of the field in the underlying database does not establish systematic ascertainment.
Clinical validity	Sufficient source evidence for reproducible clinical adjudication [8,10].	Pre-eclampsia flagged in 39 keys in the principal field and 9 in a separate field; onset week in 28 episodes. No case-level adjudication performed.	Validity dimension, separate from the three mechanisms.	Recorded labels are neither validated outcomes nor presumed false.

CS, caesarean section; MoM, multiples of the median; PlGF, placental growth factor. Full cohort N = 9008 administrative keys; principal mature cohort N = 8436. The 893 and 883 denominators are known-mode subsets within records with both later examinations. Mechanisms may coexist and are not mutually exclusive patient categories. Record-level and field-level absence are subtypes of non-documentation, not additional mechanisms. External sources justify measurement and reporting principles, not numerical benchmarking against this cohort.

**Table 2:** Temporal availability and conditional documentation across calendar epochs. (**A**) Index antenatal fields in the full cohort (*N* = 9008). (**B**) Examination and delivery fields at the principal 90-day lag (*N* = 8436). (**C**) Conditional analysis among mature keys with known delivery mode (*N* = 2387). (**D**) Outcome-maturity sensitivity.

**Panel (A).**					
**Recorded Item**	**2010–2014**	**2015–2019**	**2020–2023**	**2024–2026**	**Total**
MAP	1/1632 (0.06%)	2/2939 (0.07%)	141/2725 (5.17%)	1630/1712 (95.21%)	1774/9008 (19.69%)
PlGF MoM	0/1632 (0.00%)	0/2939 (0.00%)	3/2725 (0.11%)	20/1712 (1.17%)	23/9008 (0.26%)
**Panel (B).**					
**Recorded item**	**2010–2014**	**2015–2019**	**2020–2023**	**2024–2026**	**Total**
T2 examination	1251/1631 (76.7%)	2291/2936 (78.0%)	2147/2720 (78.9%)	834/1149 (72.6%)	6523/8436 (77.3%)
**T2 and T3 examinations**	**431/1631 (26.4%)**	**645/2936 (22.0%)**	**953/2720 (35.0%)**	**514/1149 (44.7%)**	**2543/8436 (30.1%)**
**Delivery mode**	**932/1631 (57.1%)**	**928/2936 (31.6%)**	**404/2720 (14.9%)**	**123/1149 (10.7%)**	**2387/8436 (28.3%)**
Birthweight	926/1631 (56.8%)	934/2936 (31.8%)	415/2720 (15.3%)	134/1149 (11.7%)	2409/8436 (28.6%)
Delivery indication	16/1631 (1.0%)	25/2936 (0.9%)	95/2720 (3.5%)	52/1149 (4.5%)	188/8436 (2.2%)
**Apgar at 1 min**	**837/1631 (51.3%)**	**493/2936 (16.8%)**	**120/2720 (4.4%)**	**73/1149 (6.4%)**	**1523/8436 (18.1%)**
Neonatal transfer entry	0/1631 (0.00%)	3/2936 (0.10%)	0/2720 (0.00%)	2/1149 (0.17%)	5/8436 (0.06%)
Apgar at 5 min	no field	no field	no field	no field	no field in export
Cord blood gases	0/1631 (0.0%)	0/2936 (0.0%)	0/2720 (0.0%)	0/1149 (0.0%)	0/8436 (0.0%)
**Panel (C).**					
**Recorded item**	**2010–2014**	**2015–2019**	**2020–2023**	**2024–2026**	**Total**
Indication documented	16/932 (1.7%)	25/928 (2.7%)	95/404 (23.5%)	52/123 (42.3%)	188/2387 (7.9%)
Elective caesarean label	756/932 (81.1%)	757/928 (81.6%)	277/404 (68.6%)	77/123 (62.6%)	1867/2387 (78.2%)
**Panel (D).**					
**Sensitivity measure**			**Lag 0 days**	**Lag 90 days** (**principal**)	**Lag 180 days**
Mature keys, all epochs			8618	8436	8254
Excluded from 9008, including 10 unclassifiable			390	572	754
Mature keys in 2024–2026			1331	1149	967
T2 and T3 examinations, 2024–2026			606/1331 (45.5%)	514/1149 (44.7%)	421/967 (43.5%)
Known delivery mode, 2024–2026			132/1331 (9.9%)	123/1149 (10.7%)	106/967 (11.0%)
Indication among known modes, 2024–2026			54/132 (40.9%)	52/123 (42.3%)	47/106 (44.3%)
Elective CS among known modes, 2024–2026			82/132 (62.1%)	77/123 (62.6%)	67/106 (63.2%)

Values are n/N (%). Epoch is index-examination year. Panel A uses all 9008 keys. Panel B uses the same mature denominator for later examinations and delivery fields, allowing direct comparison. The final epoch is restricted by maturity and does not represent all 2024–2026 entries. Cord gases are an investigator-confirmed structural zero within the study pathway; the 5 min Apgar has no corresponding field in the analysed export. Presence of a birthweight, Apgar or transfer entry is a documentation measure, not a clinical outcome rate. Panel C denominators are known-mode records, not the entire mature cohort; indication documentation was not adjudicated for accuracy. Panel D applies index date + (294—index gestational age in days) + lag ≤ 31 August 2026. The first three mature epoch denominators are identical across lags (1631; 2936; 2720), as are their registry-based numerators.

## Data Availability

The pseudonymised clinical dataset is held by the Maternal-Fetal Medicine Center, Timișoara, and is not publicly available because of patient-confidentiality restrictions. Aggregate data underlying the reported analyses are included in Table 1 and Table 2. Requests for additional aggregate information should be addressed to the corresponding author and remain subject to institutional approval.

## Supplementary Materials

The following supporting information can be downloaded at: https://www.mdpi.com/article/10.3390/healthcare14203526/s1: Table S1, Marker eligibility among first-trimester examinations, by calendar epoch; Table S2, Technical-review flags in the analysed cohort, by trigger; Table S3, Proposed research-specific minimum dataset for external replication, with the accompanying note on related international initiatives.
