# Supplementary material for: Can Prenatal Services Learn from Pregnancy Outcomes? A 16-Year Feasibility Study of Data Availability Across Fragmented Maternity Care
Source: Healthcare (Basel). 2026 Oct 20;14(20):3526. doi: 10.3390/healthcare14203526 (PMC13704509; doi:10.3390/healthcare14203526)
Supplement: Supplementary file 1 [file healthcare-14-03526-s001.zip › healthcare-4592888-supplementary.pdf]

Supplementary Materials

Can prenatal services learn from pregnancy outcomes? A 16-year feasibility study of data availability across fragmented maternity care

Maria Cezara Mureșan, Marius Bogdan Mureșan, Dan Dumitrașcu Biriș and Zoran Laurențiu Popa

Table S1. Marker eligibility among first-trimester examinations, by calendar epoch

Epoch	Eligible: both first-trimester markers	All singleton examinations with CRL 45–84 mm	Eligible fraction
2010–2014	1,632	3,069	53.2%
2015–2019	2,939	6,099	48.2%
2020–2023	2,725	4,896	55.7%
2024–2026	1,712	2,685	63.8%
**Total**	**9,008**	**16,805**	**53.6%**

Denominator: administrative keys with a singleton index first-trimester examination, crown–rump length 45–84 mm and examination date on or before 31 August 2026, under the same key-construction and singleton rules used for the main cohort. Numerator: keys additionally meeting the marker-eligibility criteria (PAPP-A 0.01–20 MoM and uterine artery pulsatility index 0.1–10 MoM). The numerator column reproduces the main-cohort epoch sizes exactly. Cohort entry was therefore not a constant filter across calendar time; see Section 4.7.

Table S2. Technical-review flags in the analysed cohort, by trigger

Trigger recorded in the processing issue log	Entries
Another eligible key for the same woman on the same index date	33
Reported birth date incompatible with the index examination date	15
Missing or zero index gestational age	10
Visit-chronology alert	9
More than one eligible first-trimester row for the key	7
**Total entries**	**74**

Seventy-three of the 9,008 administrative keys carried at least one open entry; one key carried two, giving 74 entries in total. Categories are not mutually exclusive at key level and must not be summed as distinct keys. These flags were generated by the analysis pipeline during construction of the derived registry; they are not statuses recorded in the clinical database and are not confirmed duplicate pregnancies. Flagged keys were retained in the cohort without merging, exclusion or unverified reconciliation, and all entries remained open at the time of analysis. The ten keys with missing or zero index gestational age are the same ten keys excluded as unclassifiable from maturity-restricted analyses; that exclusion follows from the positive-gestational-age condition of the maturity rule and not from the technical-review status.

Table S3. Proposed research-specific minimum dataset for external replication

Domain	Required elements
Identification	Verified pregnancy identifier linked to the mother; birth institution.
Dating	Dating method and index gestational age.
First-trimester markers	Marker values with acquisition and assay metadata.
Later fetal observation	Two timed later examinations with technically interpretable fetal assessments.

Domain	Required elements
Maternal phenotype	Maternal conditions of the index pregnancy, with supporting information, recorded as outcomes and not only as booking history.
Labour	Actual labour onset and exposure, recorded independently of delivery mode.
Delivery	Delivery timing and mode; structured indication with the relevant cardiotocographic or clinical evidence.
Neonatal acid–base	Paired cord-gas sampling information where required by the endpoint.
Neonatal outcomes	Defined neonatal outcomes with their assessment times, including the 5-minute Apgar score.
Provenance	Source of each outcome, date of receipt, date of entry, and verification status.
Explicit states	Distinguishable states for unknown, not performed and not returned, with the reason for an unavailable required measurement where known.

This is an author-derived recommendation for future replication, not a validated standard and not a consensus instrument. It was derived from the absences documented in the present audit and has not been evaluated in practice.

Note to Table S3. Relation to existing international initiatives

International initiatives provide useful design comparators rather than numerical benchmarks for this private antenatal cohort. The ICHOM pregnancy and childbirth proposal defines a patient-centred set spanning clinical and reported outcomes with associated case-mix factors [17]. The international pre-eclampsia core outcome set similarly shows how maternal and offspring outcomes can be agreed through a formal consensus process [18]. These frameworks support explicit definitions and timing but do not, by themselves, ensure that delivery information reaches an antenatal registry.

The Australian Perinatal National Minimum Data Set illustrates a further implementation layer: agreed data elements for the mother and baby, including labour and birth information, embedded in a defined reporting system [19]. Such a dataset is not identical to the research-specific observations needed here, nor is it evidence that every required endpoint is routinely available in every participating setting. Its relevance is the explicit specification of variables, units, permissible categories and collection responsibilities. One element shows how such specifications govern what can later be reconstructed: that dataset makes the 5-minute Apgar score a mandatory element and contains no element for the 1-minute score, the inverse of the export analysed here.

The research protocol of any future biological study must specify the primary outcome, acid–base thresholds, sampling quality checks, treatment of absent labour, adjudication rules, follow-up horizon and confounding strategy before modelling. If objective confirmation remains unavailable, a clinical-adjudication study may be feasible, but it would answer a separately defined question. No change to clinical care solely to enable research is justified by this availability audit; the proposals above concern the capture and recovery of information.

Reference numbers correspond to the reference list of the main manuscript.
